# Biatrial tachycardia due to conduction recovery of left atrial anterior roof lesion created by vein of Marshall ethanol infusion

**DOI:** 10.1016/j.jccase.2026.01.006

**Published:** 2026-04-14

**Authors:** Shushi Nishiwaki, Satoshi Shizuta, Hirohiko Kohjitani, Koh Ono

**Affiliations:** Department of Cardiovascular Medicine, Kyoto University Graduate School of Medicine, Kyoto, Japan

**Keywords:** Atrial tachycardia, Ethanol infusion, Vein of Marshall

## Abstract

A 77-year-old man underwent pulmonary vein isolation and cavotricuspid isthmus ablation for paroxysmal atrial fibrillation and typical atrial flutter. Eleven months later, recurrent atrial fibrillation required a second procedure. Left atrial (LA) posterior wall isolation and vein of Marshall (VOM) ethanol infusion were performed for roof-dependent atrial tachycardia (AT) and Marshall bundle (MB)-related triggers. A third procedure was required for recurrent AT. After completion of the anterior mitral line, an induced MB-related AT was terminated by VOM ethanol infusion, and the LA anterior roof area appeared electrically isolated. However, two weeks later, a rapid AT recurred and a fourth procedure revealed conduction recovery across the previously isolated LA anterior roof area, resulting in a counter-clockwise biatrial tachycardia. Radiofrequency ablation at the LA ridge and anterior wall terminated the AT and re-isolated the LA anterior roof area. This case demonstrates that lesions created by VOM ethanol infusion, particularly those extending toward the LA roof, may not always be durable.

**Learning objective:**

Vein of Marshall (VOM) ethanol infusion is widely used to eliminate Marshall bundle-related atrial tachycardia. When VOM ethanol infusion creates isolation extending toward the left atrial anterior roof, additional endocardial radiofrequency ablation should be considered to ensure durable block and prevent arrhythmia recurrence.

## Introduction

Atrial tachycardia (AT) is frequently encountered after atrial fibrillation (AF) ablation. Most macroreentrant ATs involve roof-dependent or perimitral circuits, whereas Marshall bundle (MB)-related ATs account for up to 30% of post-AF ablation left ATs [1]. Treatment typically involves eliminating conduction through MB-left atrium (LA) and coronary sinus (CS)-MB connections by radiofrequency (RF) ablation and/or ethanol infusion into the vein of Marshall (VOM). VOM ethanol infusion is known to create extensive epicardial injury along the mitral isthmus and its adjacent structures; however, its acute effects are not always irreversible, and lesion durability may vary depending on anatomical factors such as VOM branching patterns [2], [3], [4].

Previous studies have described the chronic durability of VOM-related lesions within the mitral isthmus, yet the persistence of lesions that extend superiorly toward the LA roof remains unclear. Here, we report a case in which the LA anterior roof appeared isolated immediately after VOM ethanol infusion but subsequently exhibited conduction recovery in the chronic phase, resulting in a biatrial tachycardia (BiAT). To our knowledge, this is the first documented case demonstrating conduction recovery specifically in an LA anterior roof lesion created by VOM ethanol infusion.

## Case report

A 77-year-old man without structural heart disease initially underwent pulmonary vein isolation (PVI) using cryoballoon and cavotricuspid isthmus ablation for paroxysmal AF and typical atrial flutter. Eleven months later, symptomatic AF recurred and was documented on 24-hour Holter monitoring, requiring a second ablation procedure. During this procedure, all pulmonary veins (PVs) were confirmed to remain electrically isolated. Because a roof-dependent AT was induced, LA posterior wall isolation was performed. AF was subsequently triggered by premature atrial contractions originating from the MB, and 3 ml of 98% ethanol was infused into the VOM using a 2.0-mm over-the-wire (OTW) balloon (Emerge, Boston Scientific, Marlborough, MA, USA). A post-infusion voltage map during right atrial (RA) pacing demonstrated a low voltage area at the LA ridge between left atrial appendage (LAA) and left inferior PV.

**Fig. 1:**
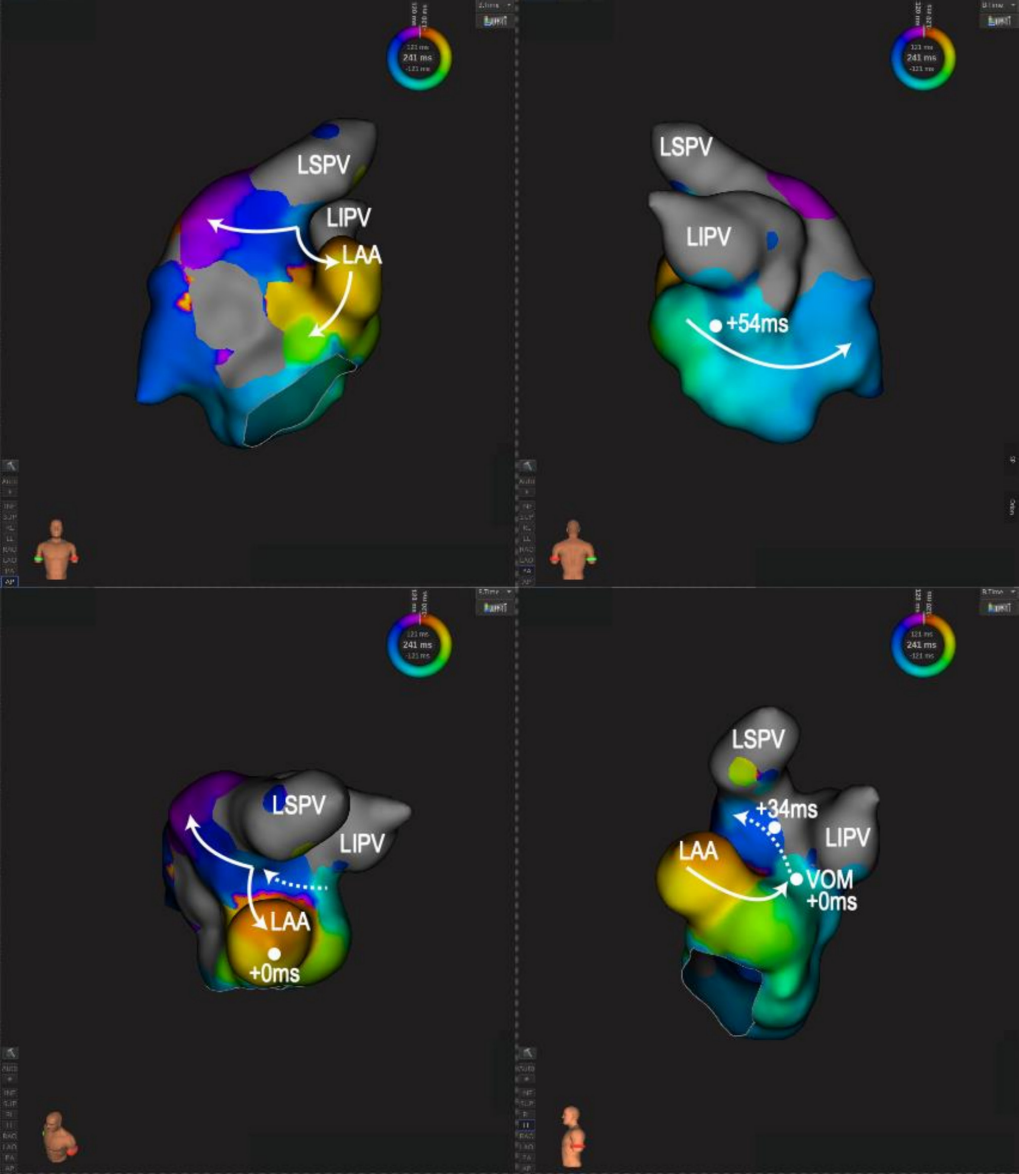
Activation maps of AT-2 demonstrating downward activation along the LAA and upward activation along the LA ridge. Solid white arrows indicate endocardial activation, and dotted white arrows indicate possible epicardial conduction via the Marshall bundle. White circles and numbers represent entrainment pacing sites and post-pacing interval minus TCL. AT, atrial tachycardia; LA, left atrial; LAA, left atrial appendage; LIPV, left inferior pulmonary vein; LSPV, left superior pulmonary vein; TCL, tachycardia cycle length; VOM, vein of Marshall.

**Fig. 2:**
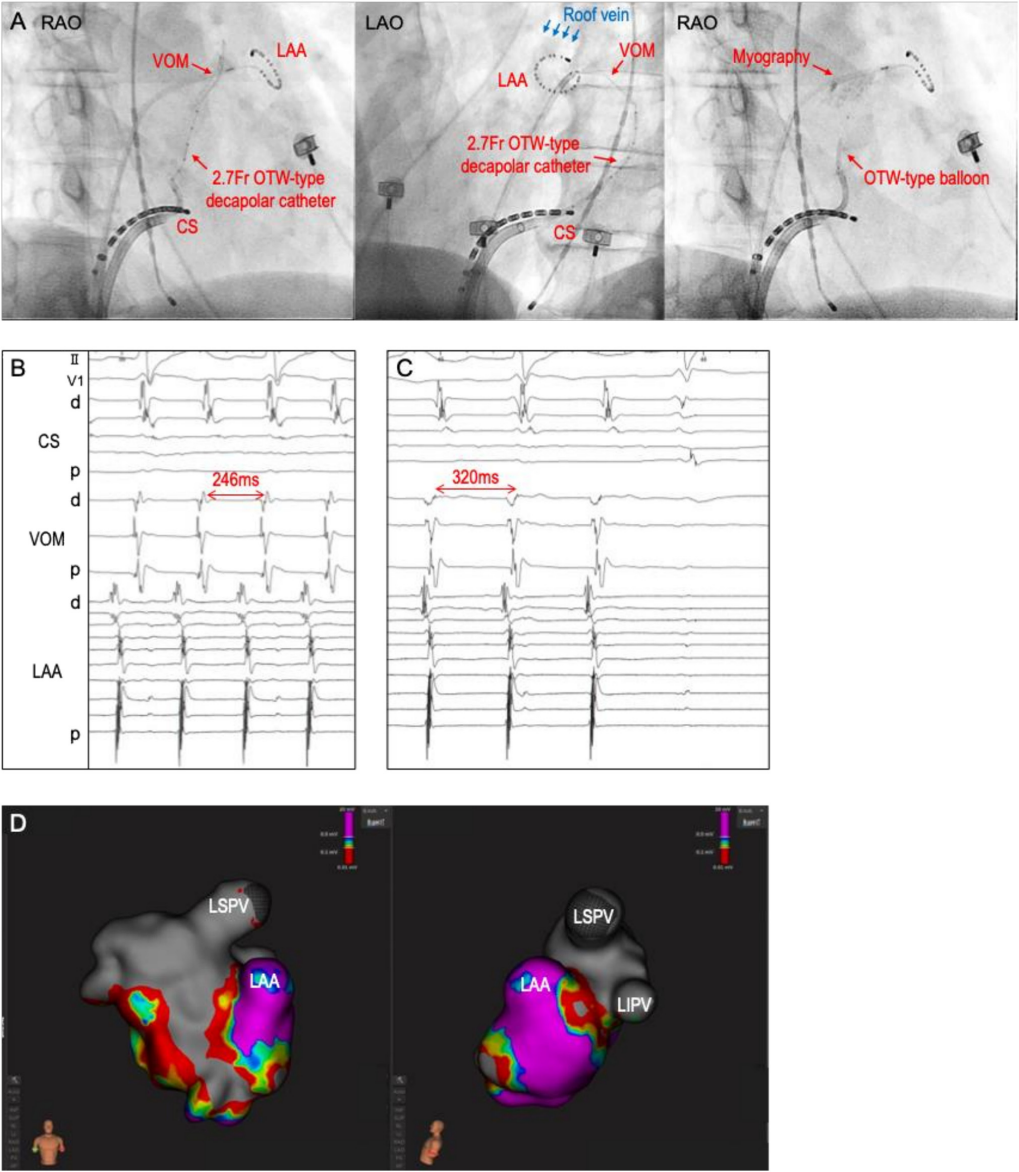
(A) Fluoroscopic images showing the position of a 2.7-Fr OTW-type decapolar catheter within the VOM and retrograde venography demonstrating catheter wedging at the distal VOM (left panel), an anastomotic connection between the VOM and a small roof vein (middle panel), and LA myography obtained during contrast injection through an OTW-type balloon (right panel). (B) Intracardiac electrocardiograms of AT-2 with TCL of 246 ms. (C) AT-2 terminated after prolongation of TCL to 320 ms during VOM ethanol infusion. (D) Voltage maps during right atrial pacing show complete isolation of the LA anterior roof area and preserved voltage in the posterolateral mitral isthmus. AT, atrial tachycardia; CS, coronary sinus; LA, left atrial; LAA, left atrial appendage; LIPV, left inferior pulmonary vein; LSPV, left superior pulmonary vein; OTW, over-the-wire; TCL, tachycardia cycle length; VOM, vein of Marshall.

Nine months after the second procedure, the patient reported frequent palpitations and presyncope. One-week Holter monitoring captured recurrent AT with long pauses (up to 10 s) at termination. The patient declined pacemaker implantation and underwent a third ablation procedure. During the third procedure, AT-1 with an eccentric CS sequence pattern was induced; however, the tachycardia cycle length and activation sequence changed frequently, preventing adequate activation mapping. AT-1 eventually degenerated into AF and was terminated by cardioversion. A voltage map during RA pacing (Rhythmia, Boston Scientific) obtained with a 64-minielectrode basket mapping catheter (IntellaMap Orion, Boston Scientific), revealed durable LA posterior wall isolation and a low voltage area along the LA anterior wall. Based on these findings, AT-1 was suspected to be a perimitral AT, and an anterior mitral line connecting the mitral annulus and right superior PV was created. Bidirectional block across the anterior mitral line was confirmed by activation mapping during LAA pacing and differential pacing. Following this, atrial burst pacing reproducibly induced AT-2. Activation mapping showed a downward activation along the LAA and upward activation along the LA ridge (Fig. 1). A 2.7-Fr OTW-type decapolar catheter (EPstar Fix AIV, Japan Lifeline, Tokyo, Japan) was advanced into the VOM (Fig. 2A, B). Entrainment pacing demonstrated that the VOM was part of the reentry circuit of AT-2. Contrast injection through the decapolar catheter showed that the catheter tip was wedged distally and revealed an anastomotic connection between the VOM and a small roof vein (Fig. 2A). Thereafter, 2 ml of 98% ethanol was infused through the decapolar catheter, resulting in termination of AT-2 (Fig. 2C). An additional 3 ml of ethanol was then injected using a 2-mm OTW balloon (Emerge) at the distal VOM to strengthen the lesion set (Fig. 2A). A subsequent voltage map during RA pacing showed complete electrical isolation of the LA anterior roof area, including Bachmann bundle (Fig. 2D). No further sustained ATs were inducible.

**Fig. 3:**
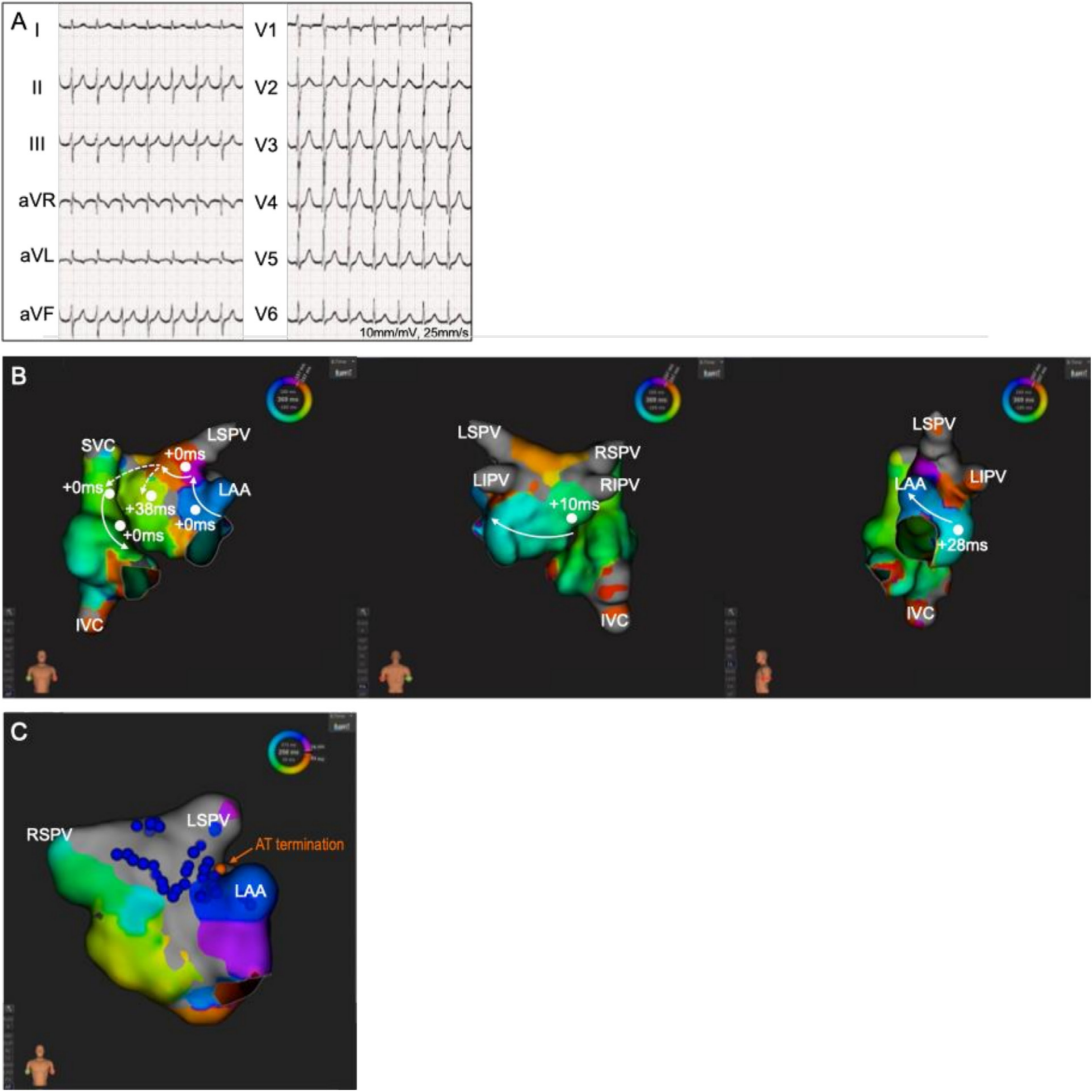
(A) Twelve-lead electrocardiogram of AT-3. (B) Activation maps demonstrating a counter-clockwise biatrial tachycardia. Solid white arrows represent endocardial activation, and dotted white arrows represent possible epicardial activation via the Bachmann bundle. White circles and numbers denote entrainment pacing sites and post-pacing interval minus TCL. (C) Activation map during LAA pacing showing re-isolation of the LA anterior roof after endocardial radiofrequency applications. Orange tag indicates the site of AT termination, and blue tags indicate sites of endocardial radiofrequency application. AT, atrial tachycardia; IVC, inferior vena cava; LA, left atrial; LAA, left atrial appendage; LIPV, left inferior pulmonary vein; LSPV, left superior pulmonary vein; RIPV, right inferior pulmonary vein; RSPV, right superior pulmonary vein; SVC, superior vena cava; TCL, tachycardia cycle length.

Two weeks after the third procedure, a rapid AT (AT-3) of 157 bpm was recorded on 12‑lead electrocardiography (Fig. 3A), and a fourth ablation procedure was performed. Activation mapping revealed conduction recovery across the previously isolated LA anterior roof, enabling a counter-clockwise perimitral AT or BiAT (Fig. 3B). Entrainment maneuvers confirmed BiAT (Fig. 3B). The first RF application delivered at the top of LA ridge terminated AT-3. Additional RF applications were delivered along the LA ridge and anterior wall. After these RF applications, activation mapping during LAA pacing demonstrated persistent conduction via the Bachmann bundle. RF applications were delivered along the anterior mitral line; however, the LA anterior roof potentials were not delayed. Targeting the breakthrough site at the LA anterior roof with RF ablation successfully re-isolated the region (Fig. 3C). Following this, neither AF nor AT was inducible with atrial burst pacing.

## Discussion

BiAT and MB-related ATs may occur after creation of an anterior mitral line, even when bidirectional block is initially confirmed [5]. VOM ethanol infusion is an effective treatment for MB-related AT, given its ability to ablate MB-LA connections and facilitate mitral isthmus block. More than half of MB-related ATs terminated with VOM ethanol infusion alone [6]. In addition, VOM ethanol infusion can create extensive epicardial injury along the mitral isthmus and is often used as an adjunct to improve the likelihood of achieving a durable mitral isthmus block [7]. In the present case, VOM ethanol infusion during the third ablation procedure successfully terminated AT-2 and resulted in an apparently complete isolation of the LA anterior roof region, including the Bachmann bundle. Notably, no low-voltage area was observed in the posterolateral mitral isthmus thereafter, consistent with targeted ethanol delivery to the distal VOM, which minimizes the risk of LAA isolation (Fig. 2D).

However, during the fourth ablation procedure, conduction recovery across the LA anterior roof was evident, enabling a macroreentrant BiAT (Figs. 2D, 3B). To the best of our knowledge, this is the first case report demonstrating conduction recovery specifically in the LA anterior roof lesion created by VOM ethanol infusion. The durability of ethanol-induced lesions may depend on several factors. First, lesion depth and transmurality are influenced by the volume of ethanol delivered. Previous studies have recommended sequential infusion of 3-ml aliquots until LA myography is observed, typically resulting in total volumes of 4–10 ml [2], [8], [9]. In the present case, although LA myography appeared after the second infusion (Fig. 2A), the total volume delivered (5 ml) may have been insufficient to produce a durable transmural lesion along the LA roof.

Second, the anatomical characteristics of the VOM and its branches significantly affect lesion distribution [3]. In the present case, venography revealed an anastomosis between the VOM and a small roof vein, suggesting that the ethanol may have preferentially dispersed toward the LA anterior roof area rather than creating uniform epicardial injury. While this vascular connection likely contributed to the initial LA anterior roof isolation, it may also have diluted the effect of VOM ethanol infusion, predisposing the region to incomplete or non-transmural injury.

Third, VOM ethanol infusion predominantly creates epicardial lesions. When lesion durability is required in thicker or structurally heterogeneous atrial regions, such as the anterior LA wall or Bachmann bundle, epicardial-only ablation may be insufficient. Even though the LA anterior wall is generally thinner than the lateral wall [10], the epicardial approach alone may not guarantee complete and lasting transmurality. This limitation may explain why previous studies have shown that combining VOM ethanol infusion with endocardial RF ablation enhances both acute success and long-term durability of mitral isthmus block, despite occasional chronic reconnection [4].

In the present case, conduction recovery at the LA anterior roof resulted in BiAT only two weeks after ethanol infusion, demonstrating the variability of lesion stability in this region. This case highlights that when VOM ethanol infusion creates isolation extending toward the LA anterior roof, additional endocardial RF ablation should be considered to ensure durable block and prevent arrhythmia recurrence.

## Consent statement

Written informed consent was obtained from the patient.

## Declaration of competing interest

None.
